# Distal Radius Extra-Articular Fractures: The Impact of Anatomical Alignment on Patient’s Perceived Outcome (A Single Center Experience)

**DOI:** 10.7759/cureus.36541

**Published:** 2023-03-22

**Authors:** Ahmed H Kamal, Ossama M Zakaria, Rabab A Majzoub, Mohammed K Alrasheed, Hafiz A Babiker, El Walid F Nasir

**Affiliations:** 1 Orthopaedics, King Faisal University, Al-Ahsa, SAU; 2 Surgery, University of Shendi, Shendi, SDN; 3 Surgery, College of Medicine, Alhasa, SAU; 4 Pediatrics, King Faisal University, Hofuf, SAU; 5 Orthopaedics and Trauma, University of Khartoum, Khartoum, SDN; 6 Public Health Dentistry, King Faisal University, Hofuf, SAU

**Keywords:** dash, elderly, functional outcome, radiological parameters, distal radius fracture

## Abstract

Purpose: The effects of the anatomical alignment of distal radial extra-articular fractures on the patient's perceived outcome have been drawing much attention recently, and much controversy exists in the literature. The primary purpose of this study was to explore the relationship between the radiological parameters of reduction (radial inclination, radial length, and radial tilt) and the patient's perceived functional outcome, which was quantified using the DASH questionnaire.

Methods: The study included one hundred twenty-four patients with distal radial extra-articular fractures managed by closed reduction and casting. Their radiological (anatomical) outcome was determined by measuring the radial inclination, tilt, and length. Subjective functional outcome was quantified using the DASH score, calculated from the Arabic-translated DASH questionnaire at three months and six months after cast removal.

Results: the mean DASH score was 31.56 SD± 9.1 at three months and 29 SD± 3.89 at six months, and the acceptable radiological results for radial tilt, radial inclination, and radial length (according to McDermid's criteria for acceptable reduction) were 77.4%, 88.7% and 74.4%, respectively. There was a significant linear correlation between two radiological parameters (radial tilt and radial length) and the DASH score at three-month follow-up, which was more profound among patients under 70 years old and with diabetes mellitus. At the six-month follow-up, there was no significant relationship between the radiological parameters and the DASH score.

Conclusion: This study confirmed that radiological outcome affects the early patient-perceived outcome, with a more significant effect among patients under 70 and diabetics. Nonetheless, over time, there will be no significant relationship between the quality of reduction and patients' perceived outcomes. And this phenomenon requires further investigation.

## Introduction

Distal radius fractures (DRFs) are prevalent in menopausal women, and their incidence rises exponentially after menopause until age 65 compared to males. [1]. In every five fractures, four are female patients. Osteoporosis is the main risk factor for DRFs among the elderly; therefore, these fractures are also known as fragility fractures [2]. DRFs commonly occur after simple falls, and their presence increases the risk of other fragility fractures like hip fractures [3]. Most DRFs among elderlies are extra-articular, unlike younger individuals, who experience a higher rate of intra-articular fractures [4]. Extra-articular DRFs are often managed by closed reduction and six weeks of immobilization in a cast when acceptable reduction criteria have been met [5]. Radial length (>5mm), radial angulation or tilt (< 15° dorsal or 20° volar), and radial inclination (>15°) are among the radiological markers for acceptable reduction [6-11].

Traditionally, grip strength and wrist joint range of motion are used as metrics to measure the functional progress that has been made after DRF treatment. However, these objective measures do not reflect the patient’s perceived outcome [12,13]. Therefore, patient-reported outcome (PRO) tools were developed to measure and quantify patients perceived outcomes following treatment, such as patient-reported wrist evaluation (PRWE) and the disability of arm, hand, and shoulder (DASH) questionnaires [12-14].

According to numerous studies, good radiographic (anatomical) reduction parameters positively affect the patient's perceived outcome [15]. Therefore, these findings favor surgical management for distal radial extra-articular fractures to achieve more anatomical reduction. Other studies, however, reported no discernible difference between the results of operative versus conservative treatment of DRFs in the elderly [16]. Moreover, surgical treatment for DRFs has its own financial and medical burdens, especially in under-resourced countries.

Published literature is scarce regarding the management of DRFs among Sudanese patients. This study sought to evaluate the impact of radiological parameters of acceptable reduction on the patient-perceived outcomes among Sudanese following conservative treatment of extraarticular DRFs.

## Materials and methods

Study design

This prospective observational Hospital-based study included a cohort of patients with DRFs. It was conducted from September 1, 2013 to September 1, 2016 in the orthopedic department at EL-Mack Nimr University Hospital in Shendai city, Sudan. The hospital had 200 beds and was the only hospital that provided orthopedic services in the city.

Study population and sample size

**Figure 1:**
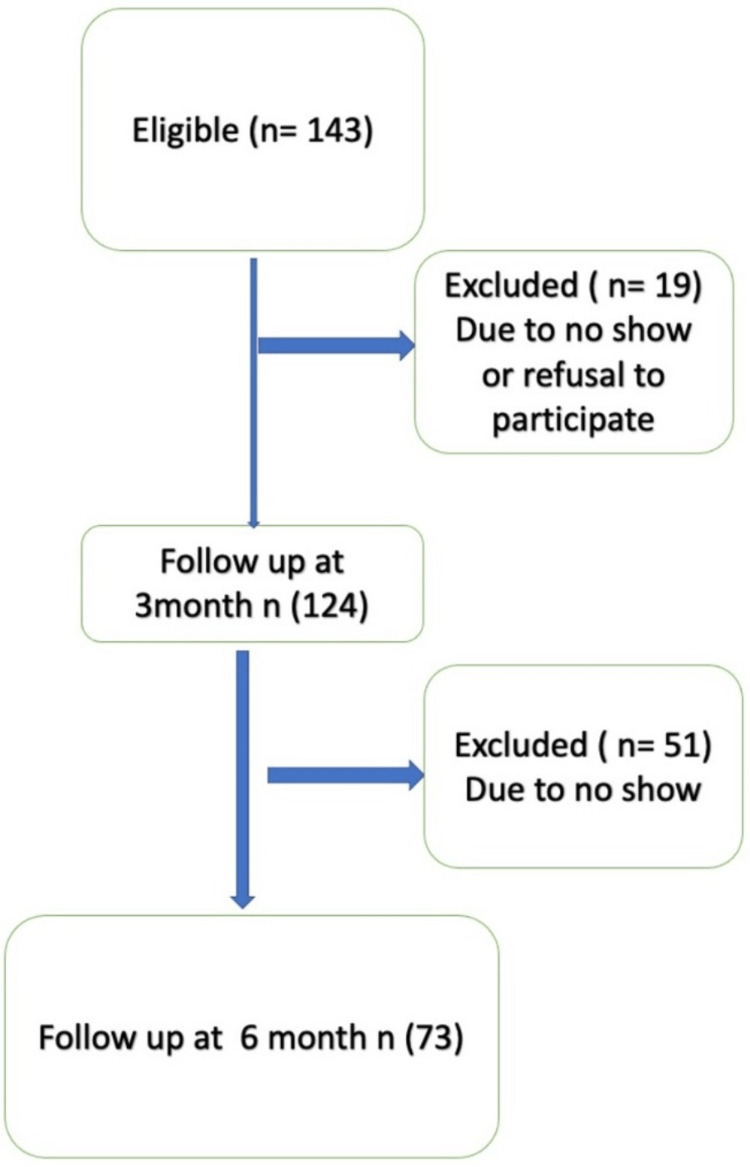
Study flow chart.

All patients aged 50 years and above with distal radius extra-articular fractures managed with closed reduction and cast application were included during the study period. All patients were treated by closed reduction and cast immobilization. Post-reduction x-ray was done immediately to check reduction quality, and any patient who had loss reduction or required re-manipulation during the six-week follow-up was removed from the study. Patients with distal radial fractures with articular extension, open fractures, bilateral fractures, and patients managed operatively were excluded. Initially, 143 patients who met the inclusion criteria were invited to the study. Their contact information was registered at the time of cast removal (an average of six weeks from the trauma after clinical and radiological evidence of union). However, only 131 patients returned to the referred clinic after three months for follow-up, and of those, only 124 patients agreed to be enrolled in the study. Of the 124, only 73 returned for follow-up at six months (Figure 1).

Data collection

Patients’ Demographics

Patients' age, gender, mode of trauma, affected limb, the timing of the initial management, fracture classification (according to AO, “Arbeitsgemeinschaft für Osteosynthesefragen” classification), and co-morbidities were all recorded in the datasheet.

Radiological Parameters

**Figure 2:**
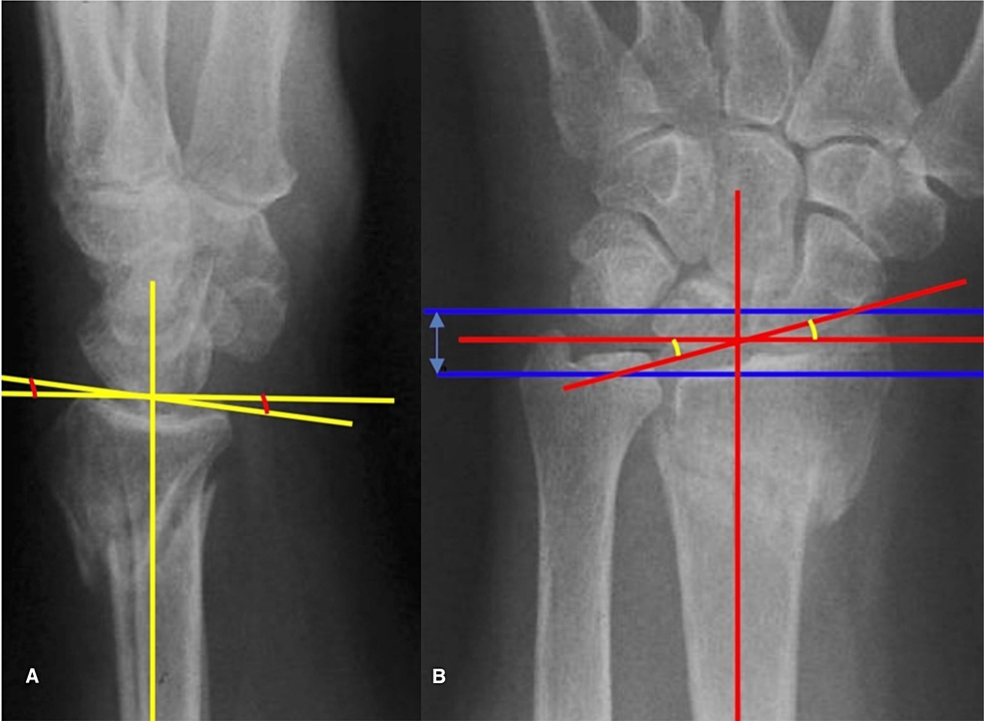
Radiographic measurements of the distal radius. (A) Lateral view of the wrist joint and the yellow lines for the measurements of Radial tilt. (B) Anteroposterior view of the wrist joint. Blue lines are for measurement of the radial length, red lines for the measurement of radial inclination.

Standard anteroposterior (AP) and lateral wrist x-ray views were used to determine the radiological parameters of reduction in order to assess the anatomical outcome; all x-rays were performed by three senior radiology technicians with experience ranging from five to 10 years. Two orthopedic surgeons with a minimum experience of five years and one senior orthopedic trainee measured the radial length, radial inclination, and radial tilt using a standard ruler and a goniometer (Figures 2A, 2B). All raters independently measured the same x-ray films for every patient included in the study. Interclass correlation coefficients were used to assess interrater reliability. Moreover, the radiological (anatomical) outcome was classified according to McDermid’s criteria for acceptable reduction into acceptable and unacceptable for each parameter [11].

Patients Perceived Outcomes

Patients reported functional outcomes were assessed using the Arabic-translated version of the DASH questionnaire, which has 30 items and a 5-point Likert scale for each item. The DASH score must also be calculated using a minimum of 27 responses; higher values denote more disability [17].

Ethical considerations

Informed consent was obtained from all individual participants included in the study. The research ethics committee of Shendi University provided its institutional permission for the research protocol. The procedures used in this study adhere to the tenets of the Declaration of Helsinki.

Statistical analysis

The data were analyzed using IBM SPSS version 25, JASP version 0.16, and Microsoft Excel 2019 edition. For categorical variables, frequency and percentages tables were used. The Kolmogorov-Simirove test of normality revealed that all variables were normally distributed; therefore, means and standard deviations (SD) were presented. Inferential statistics were done using the following tests: for the relationships between the continuous variables, Person’s correlation was used, independent samples t-test and two-way ANOVA were used for the relationship between continuous and categorical variables, and mixed ANOVA for repeated measurements was used to assess the change in DASH score between follow-ups. A p-value of less than 0.05 and a confidence Interval of 95% were considered significant.

## Results

Demographics

The mean age was 68.73 SD ±9 years, and 58.1% were below 70 years old. Most patients, 79%, were females, 62.9% of fractures involved the dominant hand, and most injuries were reported as secondary to a simple fall 93.5%. Of most patients, 72.6% received management less than 24 hours from the trauma, and according to the AO classification, the most frequently encountered type of fracture, 82.3%, was A2. The most frequent comorbidities were diabetes mellitus 35.5% and hypertension 22%, respectively (Table 1).

**Table 1:** The frequency and percentages of various patient demographics. N=124

Variable	Frequency		Percent
Gender			
Male	26		20.968
Female	98		79.032
Mode of Trauma			
Other	8		6.452
Simple fall	116		93.548
limb			
Dominant	78		62.903
Non-Dominant	46		37.097
AO classification			
A2	102		82.258
A3	22		17.742
Time of mang.			
< 24 hours	90		72.581
>24 hours	34		27.419
Age70			
<70	72		58.065
>70	52		41.935
DM			
Yes	44		35.484
No	80		64.516
Hypertension			
Yes	28		22.580
No	96		77.419

Using t-test for independent samples, patients who were less than 70 years old showed significantly higher DASH mean scores (Mean = 33.81, SD = 10.71) compared to patients who were more than 70 years old (Mean= 28.46, SD = 4.78); t(104) = 3.74, p = 0.01 with moderate effect size (Cohens d =0.67) according to Cohen's convention for effect size [18]. Furthermore, diabetic patients reported significantly high DASH mean scores (Mean = 36.6, SD = 11.1) compared to non-diabetics (Mean = 28.5, SD = 6.6); t (60) = 4.42 with large effect size (d=0.87). Other demographic variables did not differentiate in DASH scores among different groups.

Radiological parameters and DASH score at three months (N = 124)

The intraclass correlation coefficients (ICC) were used to assess inter-observer reliability utilizing a two-way mixed model and mean ratings (ICC 3,1) for absolute agreement. The ICC estimates and 95% confidence intervals (CI) of the radiological parameters were as follows: For radial length, the ICC = 0.96 with 95% CI (0.71-0.98); p-value (<0.001); for Radial inclination, the ICC = 0.92 with 95% CI (0.50-0.97); p value < 0.001, and for radial tilt the ICC = 0.82 with 95% CI (0.81-0.92), p-value < (0.001). Therefore, based on these results, the inter-rater reliability is considered excellent according to Cohen's criteria for classifying ICC values [19].

The mean DASH score was (31.56 SD± 9.1) at three months (N=124), the mean radial tilt was 0.84°±SD16.53° (volar angulation was given a positive sign while a negative sign was given to dorsal angulation), the mean radial length was (6.76 ±SD 3.36 mm), and the mean radial inclination was (19.90°±SD4.24°). According to McDermid's criteria for acceptable reduction, the radiological outcome was acceptable in (88.7%) for radial inclination, (74.2%) for radial length, and (77.4%) for radial tilt.

The correlation between the radiological measures and the DASH score was examined across all patients using Person’s correlation. Radial tilt and DASH score had a significant moderately negative (inverse)correlation (r = -0.596, p <0.001. Additionally, there was a significant negative, weak correlation (r = -0.448, p <0.001) between radial length and DASH score. Moreover, there was a weak but significant positive(direct) correlation (r = 0.256, p < 0.001) between radial inclination and DASH score. However, subgroup correlation analysis revealed a strong correlation between radial tilt and radial length with the DASH mean scores among patients younger than 70 years old. Moreover, to compare correlation coefficients between age groups, Fisher Z transformation of correlation coefficients (r) was utilized to calculate the Z statistic, which was 2.5 (p=0.006) for the radial length and 3.3 (p=0.001) for the radial tilt (Table 2).

**Table 2:** Correlation between the radiological parameters and the DASH score. N=124

			Radial length	Radial inclination		Radial tilt	
DASH All patients	Pearson Correlation	-.448**		.256**	-.595**		
	Sig. (2-tailed)	.000		.004	.000		
	N	124		124	124		
DASH >70 years	Pearson Correlation	-.437^**^		.462^**^	-.576^**^		
	Sig. (2-tailed)	.000		.000	.000		
	N	72		72	72		
DASH <70 years	Pearson Correlation	-.764^**^		.149	-.853^**^		
	Sig. (2-tailed)	.000		.292	.000		
	N	52		52	52		
Group wise Correlation Analysis	Z observed score	2.5 P = (0.006)		--	3.3 P = (0.001)		

Using the independent samples t-test, patients with unacceptable radial length and radial tilt (according to McDermid's criteria) demonstrated higher mean DASH scores, which were statistically significant (Table 3), with larger effect size (Cohen’s d) among individuals under 70 years of age (Table 4). There was no statistically significant difference in the DASH mean scores regarding radial inclination.

**Table 3:** Results of independent samples t-test between DASH score and radiological outcome. *Significant, N-124

	Acceptable	Unacceptable	t(df)	P value	Cohen’s d
Radial length	92	32	-8.24 (122)	<0.01*	-1.67
Radial tilt	96	28	-10.4(122)	<0.01*	-2.24
Radial inclination	110	14	1.12 (122)	.264	--

**Table 4:** Results of independent samples t-test between DASH score and radiological outcome in patients >70 and <70 separately. *Significant, N=124

	Acceptable	Unacceptable	Mean difference	t(df)	P value	Cohen’s d
Radial length						
>70	40	12	7.200	5.9(50)	<0.001*	-1.48
<70	50	20	15.223	6.9(70)	<0.001*	-1.82
Radial tilt						
>70	42	10	7.352	5.5(50)	<0.001*	-1.93
<70	58	18	18.778	9.9(70)	<0.001*	-2.23
Radial inclination						
>70	48	4	-3.750	-1.5(50)	0.133	--
<70	62	10	-3.723	-1.02(70)	0.311	--

A two-way ANOVA was conducted to examine the effect of interactions between patients' age groups, DM, and radiological parameters (according to McDermid's criteria) on the DASH score. There was a statistically significant interaction between the effects of patients' age and radial tilt (F (1, 120) = 19.217, p < .001.) and radial length (F (1, 120) = 7.923, p = 0.006.) on DASH score. Furthermore, there was a statistically significant interaction between the effects of DM and radial tilt (F (1, 120) = 6.315, p = 0.014.) and radial length (F (1, 120) = 4.936, p = 0.023) on DASH score. Moreover, Simple main effects analysis showed that patients < 70 and diabetic patients with unacceptable radial tilt and radial length had higher mean DASH scores compared to their counterparts. Interaction of radial inclination with patient age groups and DM did not show a significant difference in DASH mean scores.

Radiological parameters and DASH score at six months (N=73)

**Figure 3:**
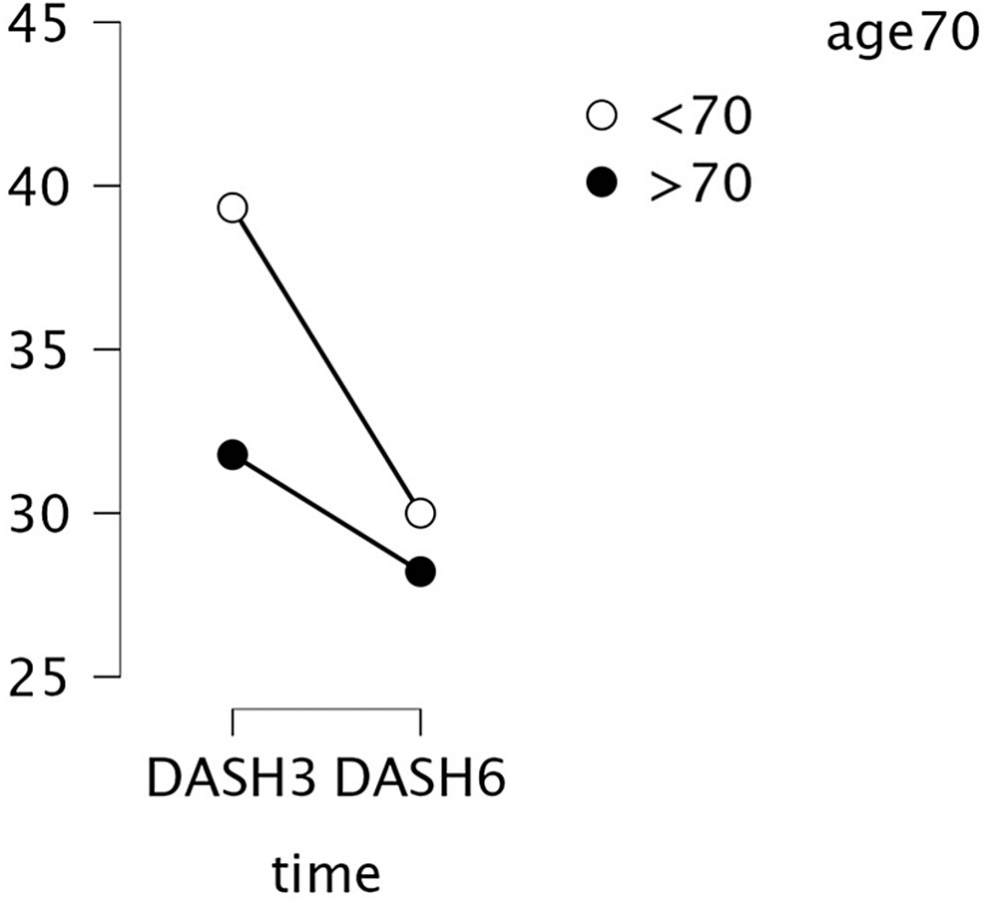
Descriptive plot showing the variation of DASH scores along time (three months, six months) among different age groups. N=73

**Figure 4:**
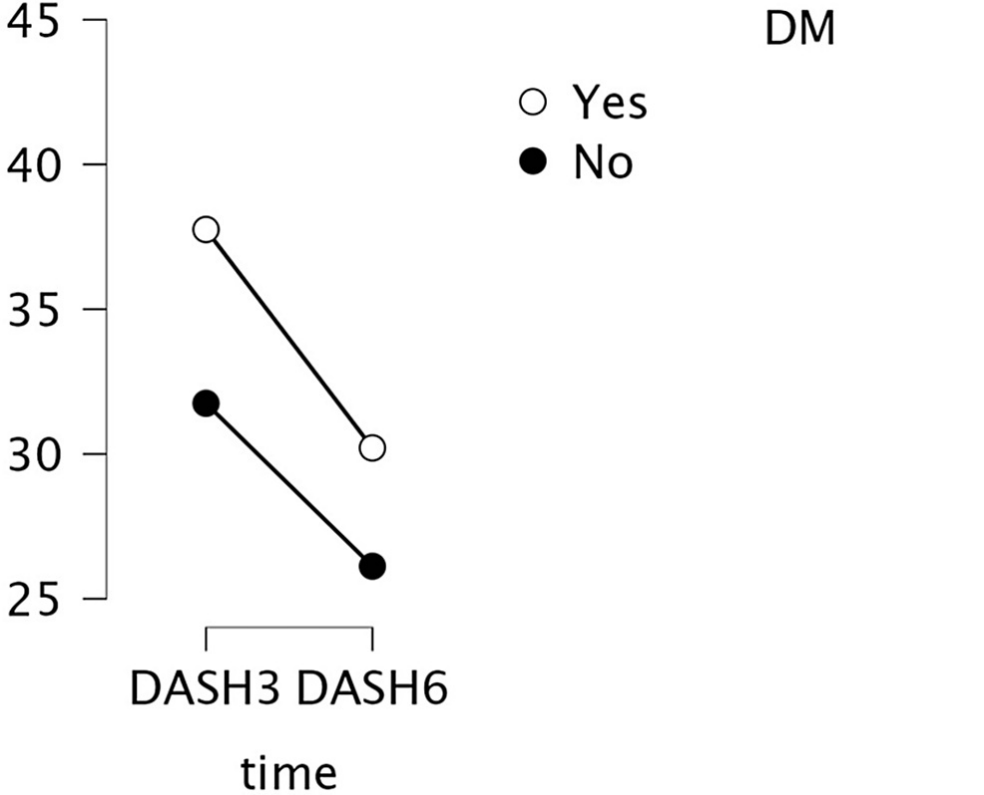
Descriptive plot showing the variation of DASH scores along time (three months, six months) among diabetics and non-diabetics. N=73

The mean DASH score for patients who returned after six months was (29 SD± 3.89). Using mixed ANOVA for repeated measurements with Greenhouse-Geisser correction, the DASH mean scores were significantly lower at six months compared to three months (F (1,72) = 69.34, p .001), with a Bonferroni adjustment for confidence interval, the mean difference was 7.12 (95% CI. 5.42 - 8.83). Additionally, patients over 70 years old and people with diabetes exhibited substantially less improvement in their DASH mean scores compared to other groups when age groups and DM diagnosis were added as fixed effects to the model (Figures 3, 4).

There was a noticeably smaller mean difference in mean DASH scores between the acceptable and unacceptable groups for the three radiological parameters at six months compared to the mean difference at three months, which was statistically significant for radial length and radial tilt (Table 5).

**Table 5:** Results of the estimated with in subject effects of time (DASH mean scores at three months and six months) and radiological parameters interactions. Using mixed ANOVA for repeated measurements. *Significant, N=73

Cases	Mean difference b/w unacceptable groups at 3-month and 6-month	df	F	p
time ✻ Radial inclination	-1.717	1	0.652	0.422
time ✻ Radial tilt	13.846	1	64.130	< .001
time ✻ Radial length	12.001	1	32.641	< .001

## Discussion

Many experts have asserted that a positive outcome is more likely when the anatomy has been correctly rebuilt after a DRF [20,21]. There is disagreement about the radiological measure that most accurately predicts the outcome. It is frequently believed that radial shortening is the most significant radiological parameter [20-22]. Nevertheless, the significance of restoring a normal palmar tilt for wrist function and carpal alignment has also been given a high value [21,23]. Since Mason's study, the radial inclination has not received much attention [24].

Although several authors have reported results supporting a correlation between radiological and patient-perceived results, others describe a lack of correlation. Our investigation demonstrated that dorsal angulation and radial shortening had a negative impact on early patient-perceived outcome (DASH), with the effect being most obvious in patients under the age of 70, and this could be explained by the higher functional demands in the younger population. And these results were in agreement with Kumar et al. [25], who also reported that patients younger than 60 had worse PRO compared to older patients. However, they did not report any changes in PRO during follow-ups.

 Our study also noted that all patients showed an improvement in PRO at six months, with younger patients showing the greatest improvement. Additionally, radiological characteristics had no discernible effect on the PRO, and these findings were consistent with those of Chang et al. [26], Anzurat et al. [27], and Gutiérrez et al. [15].

Patients with diabetes mellitus had less favorable PRO than other patients at the three-month follow-up, and diabetic patients who had unsatisfactory radial tilt and radial length reported substantially worse PRO than other patients. Although their PRO scores improved over the six months, their pace of progress was slower than that of non-diabetic patients. And these outcomes were consistent with research by Alsubheen et al. [28].

To the best of our knowledge, the impact of the three radiological parameters of reduction on the patient-perceived outcome, measured by a DASH score following extra-articular DRFs, has not been demonstrated before in Sudanese people.

One of the significant limitations of our investigation was that we lost nearly 50% of our sample by the six-month follow-up attributable to no-shows or refusals to continue the study. The follow-up period in our study was supposed to last one year. Although the DASH questionnaire was in the Arabic language, some patients found it difficult to respond properly, and we made an effort to reduce information bias by asking our junior collages to assist in filling out the questionnaire, which added to the workload, especially in busy clinic settings, this was another study limitation.

## Conclusions

In conclusion, our study confirmed that radiological outcome impacts the early patient-perceived outcome, with a more significant effect among patients under 70 and diabetics. Nonetheless, over time, there will be no significant relationship between the quality of reduction and patients' perceived outcomes. At three months, younger patients had higher DASH scores than patients over 70, which might be explained by their higher functional demands. The DASH scores of younger patients were lower after six months of follow-up, which might be attributed to a variety of factors, including the temporal effect of improved wrist mobility and physiotherapy. And in fact, further research into this phenomenon is required. Therefore, we draw the conclusion that conservative therapy is still a decent choice, especially in countries with limited resources; yet the treating doctors in the emergency department should make all efforts to achieve the highest possible acceptable reduction for fractures.
